# Transjugular Intrahepatic Portosystemic Shunt (TIPS) Outcomes for Variceal Hemorrhage During and After COVID-19: Racial, Payer, and Income Disparities — A National Retrospective Cohort Study With Stratified Sensitivity Analyses

**DOI:** 10.1016/j.gastha.2026.101052

**Published:** 2026-07-09

**Authors:** Nakul Ganju, Shubham Gupta, Sahak Hovsepian, Samay Shah, Shaunak Ganju, Farshad Aduli

**Affiliations:** 1 Department of Medicine, Howard University Hospital, Washington, District of Columbia; 2 Virtua Health, Rowan University School of Osteopathic Medicine, Camden, New Jersey; 3 Department of Internal Medicine, Kaiser Permanente Southern California, Pasadena, California; 4 Department of Internal Medicine, George Washington University Hospital, Washington, District of Columbia; 5 Sidney Kimmel Medical College, Thomas Jefferson University, Philadelphia, Pennsylvania

**Keywords:** Health Disparities, Portal Hypertension, Cirrhosis, National Inpatient Sample, Variceal Bleeding, Social Determinants of Health

## Abstract

**Background and Aims:**

Transjugular intrahepatic portosystemic shunt (TIPS) reduces mortality in esophageal variceal hemorrhage. Whether the COVID-19 pandemic worsened existing disparities in outcomes among patients undergoing TIPS remains unclear. We aimed to characterize racial, payer-based, and income-based disparities in post-TIPS outcomes before and after the COVID-19 pandemic.

**Methods:**

Using the 2016–2022 National Inpatient Sample, we conducted a retrospective cohort analysis of 31,860 weighted hospitalizations of adults with cirrhosis and esophageal variceal hemorrhage who underwent TIPS. Admissions were stratified as pre-COVID (2016–2019) and post-COVID (2020–2022). Primary outcomes were in-hospital mortality, length of stay, and total hospital charges. Multivariable regression models with COVID-19 era interaction terms were constructed; pre- and post-COVID stratified models were performed as sensitivity analyses.

**Results:**

In-hospital mortality increased from 7.8% to 9.4% post-COVID (*P* < .001). Black patients had significantly higher mortality (adjusted odds ratio [AOR]: 1.67; 95% confidence interval: 1.27–2.19) that worsened in stratified analyses (pre-COVID AOR: 1.53 vs post-COVID AOR: 1.93). Self-pay mortality risk escalated markedly post-COVID (AOR: 2.12 pre-COVID vs 3.26 post-COVID, a 54% worsening). Black patient length of stay disparity nearly doubled post-COVID (+1.71 days pre-COVID vs +3.90 days post-COVID). Native American patients emerged as disproportionately affected post-COVID (mortality AOR: 2.39; 95% confidence interval: 1.76–3.25), a group nonsignificant in the pre-COVID era.

**Conclusion:**

Structural inequities in post-TIPS outcomes persisted and worsened during the COVID-19 era, with race, payer status, and income independently predicting mortality across both eras. These findings have direct implications for equitable access to liver transplantation and underscore the need for policy reforms targeting postprocedural care coordination, insurance parity, and hospital-level accountability.

## Introduction

Transjugular intrahepatic portosystemic shunt (TIPS) remains a cornerstone in the management of portal hypertension and its complications, particularly acute esophageal variceal hemorrhage (EVH), which continues to represent one of the most severe manifestations of decompensated cirrhosis. Despite advances in endoscopic therapy, vasoactive agents, and critical care, mortality, and rebleeding remain substantial, especially among patients classified as Child-Pugh class C or class B with active bleeding.^1^

Recent evidence has reaffirmed the role of early or preemptive TIPS, performed within 72 hours of admission, in reducing rebleeding and improving survival. A 2024 individual patient meta-analysis demonstrated a hazard ratio of 0.43 (95% confidence interval [CI]: 0.32–0.60), corresponding to a 57% reduction in 1-year mortality.^2^ Other analyses report mortality reductions ranging from 32% to 47% and rebleeding risk reductions approaching 80%.^3^^,^^4^ These findings are reflected in the American Association for the Study of Liver Diseases and European Association for the Study of the Liver/Baveno VII guidelines recommending early TIPS with polytetrafluoroethylene-covered stents for patients with Child-Pugh C scores 10–13, or Child-Pugh B scores 8–9 with active bleeding.^5^^,^^6^

Despite compelling evidence, fewer than 10% of eligible patients receive early TIPS.^7^ Barriers include restricted interventional radiology availability, fragmented referral pathways, and delayed candidate identification. These system-level issues parallel broader inequities where Black and Hispanic patients and those with Medicaid experience reduced access to advanced procedures and liver transplantation.^7^ It is important to note that the present cohort consists exclusively of patients who successfully underwent TIPS; disparities observed within this already-selected population therefore represent a conservative lower bound of the total inequity across the TIPS care continuum.

The COVID-19 pandemic further amplified disparities through disruptions in procedural access, constrained ICU capacity, and workforce shortages disproportionately affecting safety net hospitals. Emerging data demonstrate racial and ethnic disparities in cirrhosis-related premature mortality during the COVID-19 pandemic.^8^

Using the 2016–2022 National Inpatient Sample (NIS), we examined trends in TIPS outcomes for EVH across demographic, socioeconomic, and hospital strata, with pre- and post-COVID stratified analyses performed to assess the robustness of findings.

## Methods

### Study Design and Data Source

This retrospective cohort study used the 2016–2022 NIS, the largest publicly available all-payer inpatient database in the United States, maintained by the Agency for Healthcare Research and Quality as part of the Healthcare Cost and Utilization Project. The NIS represents a 20% stratified sample of discharges from US community hospitals weighted to generate national estimates.

### Study Population

**Figure 1:**
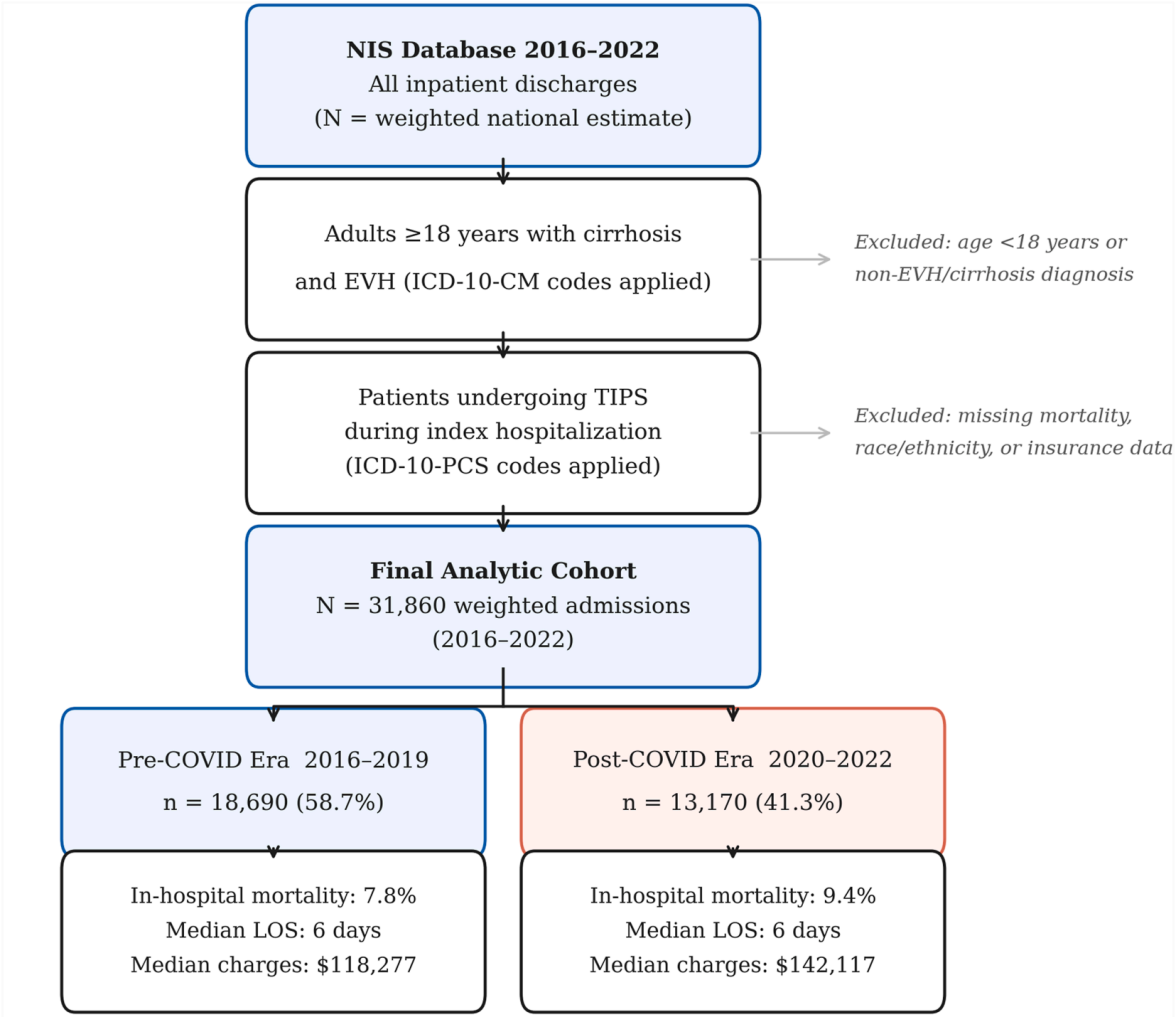
Flow diagram of study cohort selection for TIPS admissions from the National Inpatient Sample (2016–2022). CM, Clinical Modification; PCS, Procedure Coding System.

Adults ≥18 years with cirrhosis and EVH who underwent TIPS were identified using International Classification of Diseases, 10th Revision (ICD-10), Clinical Modification and ICD-10 Procedure Coding System codes (Figure 1). Records with missing mortality, race/ethnicity, or insurance data were excluded. Hospitalizations were grouped as pre-COVID (2016–2019) and post-COVID (2020–2022).

### Missing Data

Missing data were handled by listwise deletion. The final analytic sample comprised 31,860 weighted cases. Missingness for race/ethnicity was 3.0%, median household income 2.3%, and primary payer 0.2%; all other variables had <1% missing. Missingness for the primary outcome (in-hospital mortality) was 0%.

## Outcomes

Primary outcomes were in-hospital mortality, length of stay (LOS), and total hospital charges. Secondary analyses evaluated demographics, income quartile, insurance type, hospital characteristics, comorbidity burden (weighted Elixhauser Comorbidity Index), admission day, and the National Center for Health Statistics Urban-Rural Code.

### Statistical Analysis

Analyses incorporated NIS sampling weights, strata, and clusters. Non-normal continuous variables (Kolmogorov-Smirnov confirmed) were compared using the Mann-Whitney *U* test; categorical variables using the Pearson chi-square or Fisher exact test. Multivariable logistic regression identified factors associated with in-hospital mortality (adjusted odds ratio [AOR] with 95% CI). Multivariable linear regression identified predictors of LOS and charges (unstandardized coefficients B with 95% CI). Primary models were adjusted for age, sex, race/ethnicity, income quartile, payer, hospital characteristics, Elixhauser score, and calendar year, with COVID-19 era interaction terms. As prespecified sensitivity analyses to assess robustness, all regression models were repeated separately for the pre-COVID (2016–2019) and post-COVID (2020–2022) cohorts; results are presented in Supplementary Tables 1 and 2. Significance threshold: 2-sided *P* < .05. Analyses were performed using IBM SPSS Statistics v27.0.1 (IBM Corp., Armonk, NY). This study is reported in accordance with the STROBE guidelines for cohort studies.

## Results

### Patient and Hospital Characteristics

A total of 31,860 weighted admissions met inclusion criteria (Table 1). Of these, 58.7% occurred pre-COVID (n = 18,690) and 41.3% post-COVID (n = 13,170). Median age declined slightly post-COVID (58 vs 59 years; *P* = .033). Most patients were male (62%) with no temporal variation. White patients decreased from 71.5% to 70.8%, while Black patients increased from 4.1% to 4.8% (*P* = .001). The lowest income quartile fell from 33.2% to 29.4% (*P* < .001); Medicaid rose from 25.1% to 26.7% (*P* < .001).

**Table 1:** Demographic, Clinical, and Hospital Characteristics of TIPS Admissions Before and During the COVID-19 Era (2016–2022)

Characteristic	Pre-COVID (2016–2019)	Post-COVID (2020–2022)	Total, N (%)	*P* value
Age, y, mean ± SD	57.5 ± 11.7	57.1 ± 12.3	57.3 ± 12.1 (−)	.033
LOS, d, mean ± SD	8.5 ± 10.1	9.1 ± 11.9	8.9 ± 11.2 (−)	.006
Total charges, USD, mean ± SD	173,881 ± 194,187	213,810 ± 278,864	196,741 ± 247,042 (−)	<.001
Elixhauser Comorbidity index, mean ± SD	18.3 ± 7.8	18.7 ± 7.9	18.5 ± 7.9 (−)	<.001
Sex, %				.455
Male	62.6	62.1	19,849 (62.3)	
Female	37.4	37.9	12,011 (37.7)	
Race/ethnicity, %				.001
White	71.5	70.8	22,652 (71.1)	
Black	4.1	4.8	1434 (4.5)	
Hispanic	17.5	16.8	5448 (17.1)	
Asian/Pacific Islander	2.1	2.1	669 (2.1)	
Native American	1.6	1.7	510 (1.6)	
Other	3.2	3.8	1115 (3.5)	
Median household income quartile, %				<.001
0–25th percentile	33.2	29.4	9877 (31.0)	
26th–50th percentile	26.0	27.0	8475 (26.6)	
51st–75th percentile	23.3	24.9	7742 (24.3)	
76th–100th percentile	17.5	18.7	5799 (18.2)	
Primary payer, %				<.001
Medicare	38.7	36.7	11,948 (37.5)	
Medicaid	25.1	26.7	8284 (26.0)	
Private insurance	27.9	28.3	8985 (28.2)	
Self-pay	4.9	4.6	1497 (4.7)	
Other	3.3	3.9	1147 (3.6)	
Region, %				.038
Northeast	17.5	16.9	5480 (17.2)	
Midwest	20.4	20.9	6595 (20.7)	
South	40.4	39.4	12,680 (39.8)	
West	21.7	22.7	7105 (22.3)	
Hospital ownership, %				<.001
Government, nonfederal	18.5	17.1	5639 (17.7)	
Private, not-for-profit	72.5	75.4	23,608 (74.1)	
Private, investor-owned	9.0	7.5	2613 (8.2)	
Location/teaching status, %				.189
Rural	1.2	1.3	414 (1.3)	
Urban nonteaching	8.7	8.3	2708 (8.5)	
Urban teaching	90.1	90.4	28,770 (90.3)	
Weekend admission, %	16.5	18.6	5639 (17.7)	<.001
In-hospital mortality, %	7.8	9.4	2772 (8.7)	<.001

Overall N = 31,860 weighted admissions (pre-COVID n, 18,690; post-COVID n, 13,170). Continuous variables compared using the Mann-Whitney *U* test. Categorical variables compared using chi-square or Fisher exact test.

SD, standard deviation; USD, US dollars.

**Figure 2:**
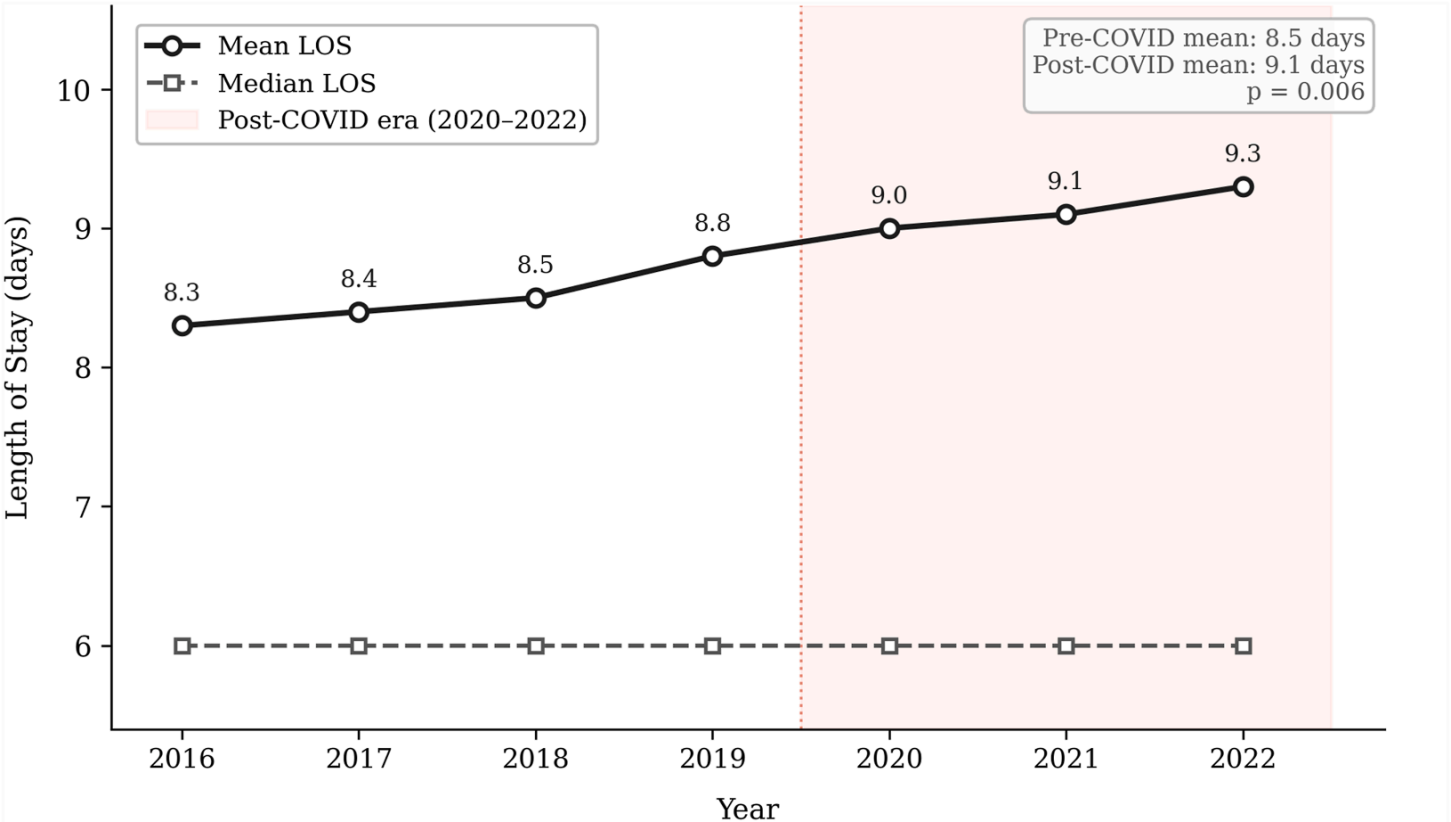
Trends in mean and median length of stay (days) among patients undergoing TIPS, 2016–2022. Median IQR: 2–11 days in both periods. Mean values labeled. Shaded area: post-COVID era (2020–2022).

**Figure 3:**
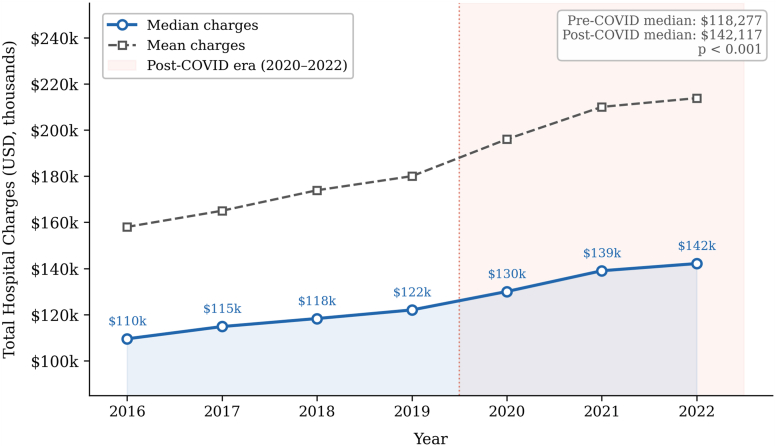
Trends in median and mean total hospital charges (USD) for TIPS admissions, 2016–2022. USD, US dollars. Median values labeled. Shaded area indicates post-COVID era (2020–2022).

Private not-for-profit hospitals performed an increasing share of TIPS (72.5%–75.4%; *P* < .001). Weekend admissions rose from 16.5% to 18.6% (*P* < .001). Mean LOS increased from 8.5 to 9.1 days (*P* = .006; Figure 2); median charges rose from $118,277 to $142,117 (*P* < .001; Figure 3). In-hospital mortality increased from 7.8% to 9.4% (*P* < .001).

### Predictors of In-Hospital Mortality

**Figure 4:**
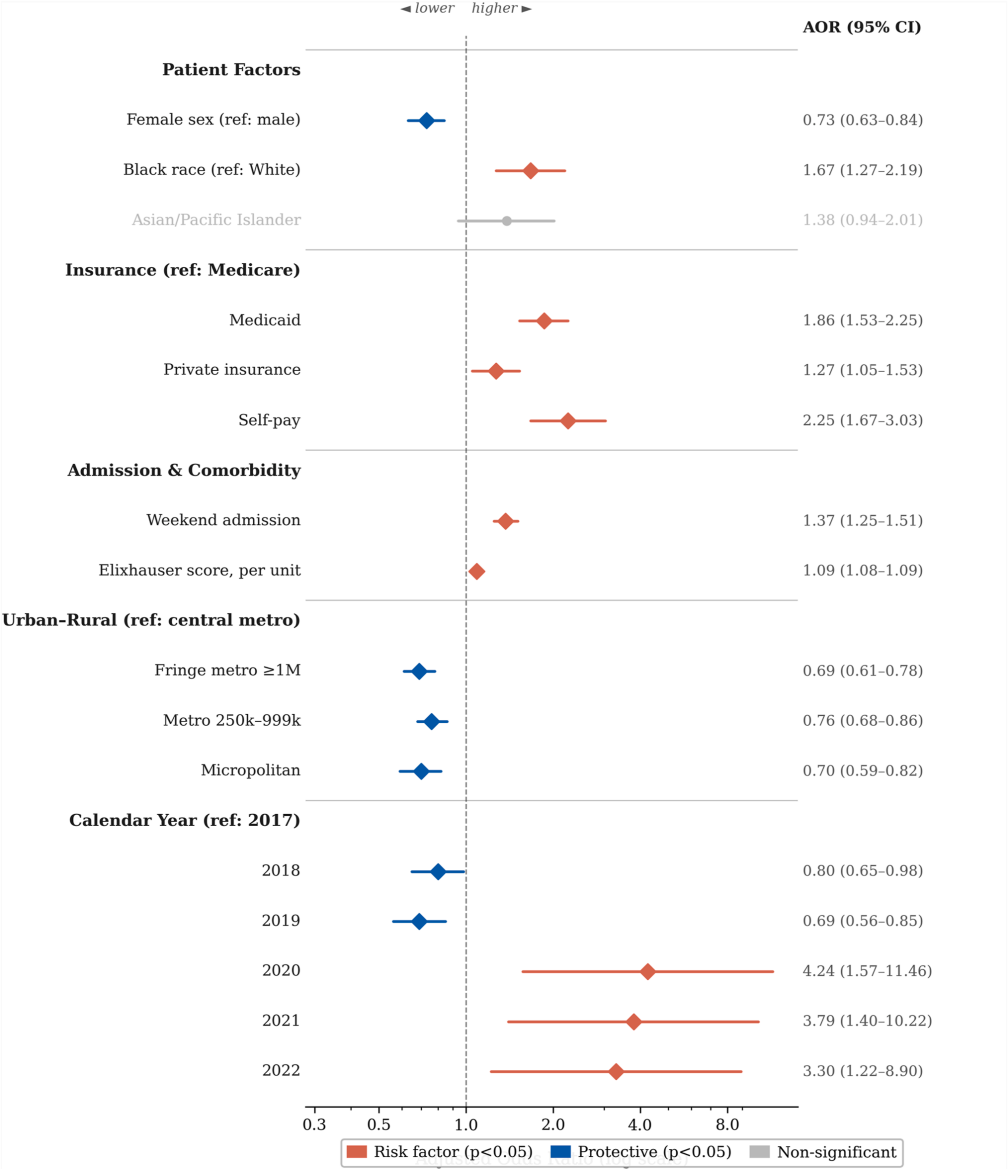
Forest plot of adjusted odds ratios for predictors of in-hospital mortality among patients undergoing TIPS, pre- and post-COVID era (2016–2022). Models adjusted for age, sex, race/ethnicity, income quartile, payer, hospital characteristics, Elixhauser Comorbidity Score, and calendar year.

**Figure 5:**
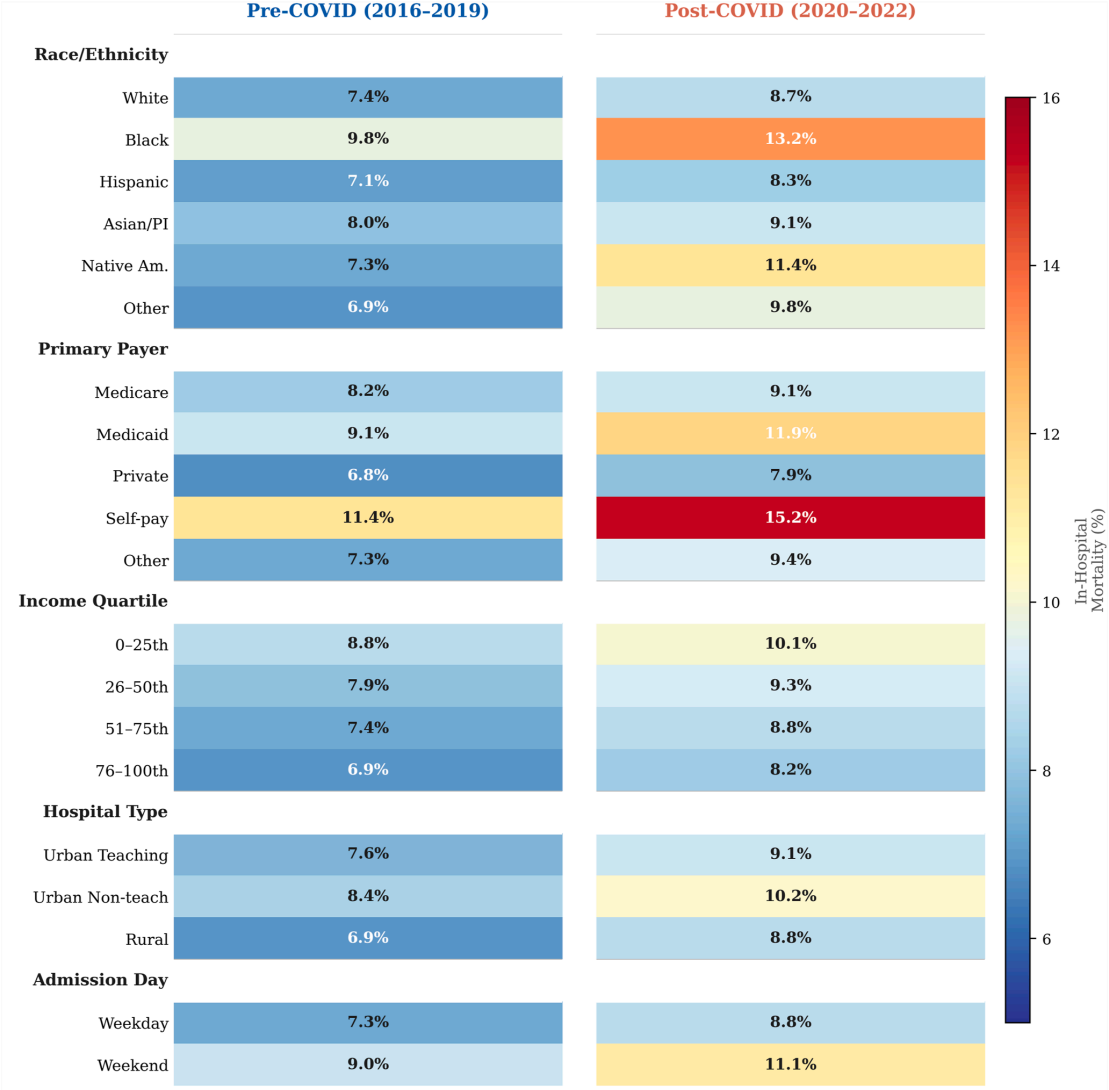
Heat map of estimated in-hospital mortality rates (%) by patient and hospital characteristics, before and after COVID-19 (2016–2022). Values represent estimated in-hospital mortality rates (%). Color scale: blue = lower mortality, red = higher mortality. Am., American; PI, Pacific Islander.

In adjusted models (Table 2, Figure 4), calendar years 2020, 2021, and 2022 were independently associated with markedly higher in-hospital mortality compared with 2017 (AOR: 4.24, 3.79, and 3.30; all *P* < .05). Each one-unit increase in the Elixhauser Comorbidity Index was associated with higher odds of death (AOR: 1.09; 95% CI: 1.08–1.09; *P* < .001). Black race was independently associated with higher mortality (AOR: 1.67; 95% CI: 1.27–2.19; *P* < .001). Medicaid (AOR: 1.86; 95% CI: 1.53–2.25; *P* < .001) and self-pay status (AOR: 2.25; 95% CI: 1.67–3.03; *P* < .001) predicted higher mortality vs Medicare. Female sex was protective (AOR: 0.73; 95% CI: 0.63–0.84; *P* < .001). Weekend admission independently predicted higher mortality (AOR: 1.37; 95% CI: 1.25–1.51; *P* < .001; Figure 5).

**Table 2:** Multivariable Logistic Regression of Factors Associated With In-Hospital Mortality After TIPS (2016–2022)

Variable	AOR	95% CI	*P* value
Age, per y	0.999	0.992–1.006	.755
Female sex (ref: male)	0.73	0.63–0.84	<.001
Race/ethnicity (ref: White)			
Black	1.67	1.27–2.19	<.001
Hispanic	0.94	0.78–1.12	.468
Asian/Pacific Islander	1.38	0.94–2.01	.098
Native American	0.92	0.56–1.49	.724
Other	0.82	0.55–1.22	.328
Income quartile (ref: 0–25th percentile)			
26th–50th percentile	1.04	0.88–1.24	.616
51st–75th percentile	0.90	0.74–1.08	.239
76th–100th percentile	0.91	0.74–1.13	.400
Primary payer (ref: Medicare)			
Medicaid	1.86	1.53–2.25	<.001
Private insurance	1.27	1.05–1.53	.014
Self-pay	2.25	1.67–3.03	<.001
Region (ref: Northeast)			
South	0.82	0.73–0.94	.003
Midwest	1.04	0.91–1.18	.602
West	0.99	0.86–1.13	.834
Urban–rural code (ref: central metro ≥1M)			
Fringe metro ≥1M	0.69	0.61–0.78	<.001
Metro 250k–999k	0.76	0.68–0.86	<.001
Metro 50k–249k	0.84	0.72–0.98	.024
Micropolitan	0.70	0.59–0.82	<.001
Non-metro/micropolitan	0.71	0.58–0.86	<.001
Weekend admission	1.37	1.25–1.51	<.001
Elixhauser Comorbidity index, per unit	1.09	1.08–1.09	<.001
Calendar y (ref: 2017)			
2018	0.80	0.65–0.98	.032
2019	0.69	0.56–0.85	<.001
2020	4.24	1.57–11.46	.004
2021	3.79	1.40–10.22	.009
2022	3.30	1.22–8.90	.018

N = 31,860 weighted admissions. Multivariable logistic regression adjusted for all variables listed. Interaction terms for COVID-19 era included.

## Predictors of Length of Stay and Hospital Charges

Predictors of prolonged LOS and higher charges are shown in Table 3. The Black race was associated with +1.85 days LOS (95% CI: 0.95–2.75; *P* < .001) and +$83,418 in post-COVID charges vs pre-COVID White patients (95% CI: $58,017–$108,819; *P* < .001). Post-COVID interaction effects revealed disproportionate LOS increases among Black (+2.0 days), Asian or Pacific Islander (+3.1 days), and Native American (+2.7 days) patients and among Medicaid (+0.98 days) and other-payer (+2.19 days) groups.

**Table 3:** Multivariable Linear Regression of Factors Associated With Length of Stay and Total Hospital Charges After TIPS (2016–2022)

Variable	Length of stay (d)			Total charges (USD)		
	B	95% CI	*P*	B	95% CI	*P*
Age, per y	−0.05	−0.07 to −0.03	<.001	−1977	−2372 to −1582	<.001
Female sex (ref: male)	+0.69	0.33–1.05	<.001	+8353	331–16,374	.041
Race/ethnicity (ref: White)						
Black^a^	+1.85	0.95–2.75	<.001	+83,418^b^	58,017–108,819	<.001
Hispanic^a^	+0.46	−0.02–0.95	.062	+20,839^b^	6887–34,791	.003
Native American	−1.15	−2.55–0.26	.110	−53,262	−84,308 to −22,216	.001
Other†	−0.24	−1.25–0.77	.639	+49,407^b^	21,123–77,692	.001
Primary payer (ref: Medicare)						
Medicaid†	+0.53	0.00–1.05	.049	+22,046^b^	6641–37,451	.005
Private insurance	+0.27	−0.20–0.75	.261	+25,651	15,084–36,218	<.001
Weekend admission	+2.08	1.78–2.38	<.001	+35,303	28,672–41,934	<.001
Elixhauser Comorbidity index, per unit	+0.48	0.46–0.49	<.001	+8805	8478–9132	<.001
Hospital: Large bed size	+1.25	0.80–1.70	<.001	+35,544	25,617–45,471	<.001
Hospital: private for-profit (ref: government)	—	—	—	+135,896	124,446–147,346	<.001
Hospital: urban teaching (ref: rural)	+2.04	0.42–3.66	.013	—	—	—

N = 31,860 weighted admissions.

B, unstandardized coefficient; USD, US dollars.

a Post-COVID (2020–2022) interaction effect versus pre-COVID reference period.

b Post-COVID (2020–2022) interaction effect vs pre-COVID reference.

### Sensitivity Analyses: Stratified Pre- and Post-COVID Models

To confirm robustness, all primary regression models were repeated separately in pre-COVID (2016–2019, n = 18,690) and post-COVID (2020–2022, n = 13,170) cohorts (Supplementary Tables 1 and 2). Stratified analyses confirmed and strengthened the primary findings, with several key observations.

For in-hospital mortality (Supplementary Table 1), Black race was a significant predictor in both eras, with risk worsening post-COVID (AOR: 1.53; 95% CI: 1.16–2.02 pre-COVID; AOR: 1.93; 95% CI: 1.58–2.37 post-COVID). Self-pay mortality risk escalated most strikingly: AOR rose from 2.12 (95% CI: 1.56–2.88) pre-COVID to 3.26 (95% CI: 2.58–4.11) post-COVID—a 54% worsening. Native American patients, nonsignificant pre-COVID (AOR: 1.03, *P* = .904), emerged as markedly high-risk post-COVID (AOR: 2.39; 95% CI: 1.76–3.25; *P* < .001). Weekend admission strengthened post-COVID (pre-COVID AOR: 1.17; *P* = .065; post-COVID AOR: 1.48, *P* < .001). Large hospital bed size shifted from protective pre-COVID (AOR: 0.69; *P* = .002) to harmful post-COVID (AOR: 1.27; *P* = .035), consistent with pandemic-related capacity strain at high-volume centers.

For LOS (Supplementary Table 2), Black patients experienced nearly doubled LOS disparity post-COVID (+1.71 days pre-COVID vs +3.90 days post-COVID; both *P* < .001). Medicaid-associated LOS increased from +0.67 days pre-COVID to +1.51 days post-COVID. Asian/Pacific Islander patients showed no significant LOS difference pre-COVID but demonstrated +2.03 days post-COVID (*P* = .001), and Native American patients similarly shifted from nonsignificant pre-COVID to +1.55 days post-COVID (*P* = .019). These stratified results confirm that the interaction effects in the primary models are consistent and reproducible.

## Discussion

This national analysis demonstrates that structural inequities in post-TIPS outcomes for EVH persisted and worsened during the COVID-19 pandemic, with stratified sensitivity analyses across both eras confirming the robustness of findings. Several patterns have direct clinical and policy relevance.

A central question raised by these findings is why outcomes differ by race, payer, and income among patients who have already successfully undergone TIPS—that is, among a population that has already cleared upstream barriers to procedural access. We propose that the answer lies largely in the post-procedural care pathway rather than the procedure itself. Successful TIPS placement is only the beginning of an intensive period of longitudinal management: patients require surveillance for hepatic encephalopathy, adherence to lactulose and/or rifaximin, nutritional optimization, and periodic Doppler ultrasound to confirm shunt patency. Each of these depends heavily on continuity of outpatient hepatology care, health literacy, transportation, and social support—domains in which Black, Medicaid-insured, and low-income patients face well-documented structural barriers. We hypothesize that gaps in this postdischarge care pathway, rather than differences in the TIPS procedure itself, are the proximate drivers of the mortality, LOS, and cost disparities observed in this cohort.

These disparities have direct implications for liver transplantation. TIPS serves as a critical bridge stabilizing patients for transplant evaluation, and persistently worse post-TIPS outcomes among publicly insured and uninsured populations may perpetuate inequities in transplant candidacy and survival.

An important alternative explanation for the temporal trends observed must be considered. The 2016–2022 study period coincides with a major shift in clinical practice toward earlier, preemptive TIPS placement following long-term follow-up data published in 2019 demonstrating a durable survival benefit with this approach.^9^ This practice shift plausibly resulted in a higher-acuity, broader population of patients undergoing TIPS in the latter years of our study period, independent of any COVID-19-specific effect. Our study design cannot fully disentangle this secular practice-pattern shift from pandemic-specific effects, as both unfolded over a similar time frame—a limitation we acknowledge explicitly. Relatedly, it is plausible that pandemic-era patient redistribution led to a higher proportion of TIPS procedures being performed at lower-volume or less experienced centers, which may disproportionately serve Black and Medicaid-insured populations. Our finding that large hospital bed size shifted from protective pre-COVID (AOR: 0.69) to harmful post-COVID (AOR: 1.27) is consistent with this hypothesis, suggesting that even high-volume centers experienced capacity strain that disproportionately affected outcomes. We frame this as hypothesis-generating and call for future hospital-volume-stratified and interrupted-time-series analyses to disentangle secular practice trends from pandemic-specific effects.

The escalation of self-pay mortality risk from AOR 2.12 pre-COVID to 3.26 post-COVID represents one of the most striking findings, suggesting that uninsured patients faced compounded vulnerability during the pandemic—likely through delayed presentation, reduced postprocedure monitoring access, and greater susceptibility to pandemic-related system disruptions. Similarly, the emergence of Native American patients as a high-risk group post-COVID—nonsignificant pre-COVID but AOR 2.39 for mortality post-COVID—is a novel finding that likely reflects the disproportionate impact of COVID-19 on Indigenous communities through compounded geographic access barriers, healthcare infrastructure limitations, and social determinants of health.

Translating these findings into policy requires moving beyond general calls for “equitable access” toward specific, implementable interventions targeting the post-TIPS care pathway. We propose: (1) bundled post-TIPS care pathways that embed patient navigators for Medicaid-insured and uninsured patients to coordinate hepatic encephalopathy monitoring and medication adherence; (2) Medicaid reimbursement parity for outpatient Doppler ultrasound surveillance of shunt patency, which is currently inconsistently covered and may contribute to delayed detection of shunt dysfunction; (3) telehepatology-based follow-up programs specifically targeting rural, Native American, and other underserved populations identified as high-risk in this analysis; and (4) hospital-level equity dashboards, modeled on existing CMS health equity reporting frameworks, that track 30-day post-TIPS readmission and mortality stratified by race and payer to drive hospital-level accountability.

Limitations include the absence of clinical parameters (Model for End-Stage Liver Disease score, hemodynamic data, and procedural timing) in the NIS, the inability to capture pre-TIPS referral patterns or postdischarge outcomes, and potential ICD-10 misclassification. As discussed above, the inability to separate secular practice-pattern shifts from pandemic-specific effects is an important limitation of any study spanning this period. These are inherent limitations of large administrative database research.

## Conclusion

Across 7 years of nationally representative data, structural inequities in post-TIPS outcomes remain profound and were amplified during the pandemic. Stratified sensitivity analyses confirm these findings are robust across both eras. Closing these gaps requires intentional, equity-focused redesign of postprocedural care pathways, reimbursement structures, and referral networks.

## Declaration of Generative AI and AI-Assisted Technologies in the Writing Process

During the preparation of this work, the authors did not use generative AI or AI-assisted technologies in the writing process.

## Supplementary Materials

Supplementary Materials

Extended PDF
