# Supplementary material for: Transjugular Intrahepatic Portosystemic Shunt (TIPS) Outcomes for Variceal Hemorrhage During and After COVID-19: Racial, Payer, and Income Disparities — A National Retrospective Cohort Study With Stratified Sensitivity Analyses
Source: Gastro Hep Adv. 2026 Jul 9;5(10):101052. doi: 10.1016/j.gastha.2026.101052 (PMC13476494; doi:10.1016/j.gastha.2026.101052)
Supplement: Supplementary file 1 [file mmc1.pdf]

Supplementary Material

TIPS Outcomes for Variceal Hemorrhage During and After COVID-19: Racial, Payer, and Income Disparities

Ganju N, Gupta S, Hovsepian S, Shah S, Ganju S, Aduli F

These supplementary tables present pre-specified sensitivity analyses in which all primary regression models were repeated separately in the pre-COVID (2016–2019) and post-COVID (2020–2022) cohorts to assess robustness of findings across both eras.

Supplementary Table S1. Sensitivity Analysis: Multivariable Logistic Regression of In-Hospital Mortality After TIPS, Stratified by Pre-COVID (2016–2019) and Post-COVID (2020–2022) Era

Variable	Pre-COVID (2016–2019)			Post-COVID (2020–2022)		
	AOR	95% CI	p	AOR	95% CI	p
**Age, per year**	0.997	0.991–1.004	0.444	0.993	0.988–0.998	0.005*
**Female sex (ref: male)**	0.720	0.624–0.830	<0.001*	0.728	0.651–0.814	<0.001*
**Race/Ethnicity (ref: White)**						
Black	1.527	1.158–2.015	0.003*	1.933	1.577–2.368	<0.001*
Hispanic	0.915	0.758–1.104	0.355	0.721	0.613–0.849	<0.001*
Asian/Pacific Islander	1.368	0.930–2.011	0.111	0.907	0.640–1.284	0.582
Native American	1.031	0.625–1.702	0.904	2.394	1.764–3.247	<0.001*
Other	0.779	0.521–1.166	0.225	1.736	1.387–2.173	<0.001*
**Income quartile (ref: 0–25th percentile)**						
26th–50th percentile	1.087	0.914–1.292	0.347	1.170	1.017–1.346	0.028*
51st–75th percentile	0.981	0.810–1.189	0.847	0.917	0.786–1.071	0.273
76th–100th percentile	1.015	0.811–1.272	0.894	1.006	0.842–1.202	0.947

**Primary payer (ref: Medicare)**						
Medicaid	1.889	1.555–2.294	<0.001*	1.662	1.417–1.948	<0.001*
Private insurance	1.288	1.064–1.559	0.010*	1.206	1.029–1.413	0.021*
Self-pay	2.120	1.561–2.880	<0.001*	3.257	2.580–4.113	<0.001*
Other	1.482	1.011–2.171	0.044*	1.628	1.245–2.128	<0.001*
**Region (ref: Northeast)**						
South	0.899	0.732–1.103	0.308	0.785	0.667–0.924	0.004*
Midwest	1.154	0.929–1.433	0.196	0.968	0.818–1.146	0.707
West	0.904	0.725–1.126	0.366	1.037	0.876–1.227	0.674
**Hospital teaching status (ref: rural)**						
Urban nonteaching	1.930	0.918–4.059	0.083	0.500	0.324–0.771	0.002*
Urban teaching	1.384	0.676–2.835	0.374	0.473	0.320–0.702	<0.001*
**Hospital bed size (ref: small)**						
Medium	0.839	0.650–1.083	0.177	1.493	1.180–1.889	0.001*
Large	0.693	0.548–0.877	0.002*	1.268	1.017–1.582	0.035*
**Urban–rural code (ref: central metro ≥1M)**						
Fringe metro ≥1M	0.491	0.401–0.602	<0.001*	0.849	0.725–0.995	0.043*
Metro 250k–999k	0.557	0.461–0.674	<0.001*	0.918	0.793–1.062	0.250
Micropolitan	0.498	0.375–0.661	<0.001*	0.851	0.690–1.050	0.133
Not metro/micropolitan	0.713	0.535–0.949	0.021*	0.672	0.513–0.882	0.004*

**Weekend admission**	1.165	0.991–1.370	0.065	1.482	1.314–1.672	<0.001*
**Elixhauser Comorbidity Index, per unit**	1.098	1.089–1.107	<0.001*	1.080	1.073–1.087	<0.001*

**p<0.05. Models adjusted for all variables listed. AOR, adjusted odds ratio; CI, confidence interval. Notable: Self-pay AOR escalated from 2.12 pre-COVID to 3.26 post-COVID (+54%). Black AOR worsened from 1.53 to 1.93. Native American patients non-significant pre-COVID (AOR 1.03, p=0.904) but markedly high-risk post-COVID (AOR 2.39, p<0.001). Large hospital shifted from protective pre-COVID (AOR 0.69) to harmful post-COVID (AOR 1.27).*

Supplementary Table S2. Sensitivity Analysis: Multivariable Linear Regression of Length of Stay After TIPS, Stratified by Pre-COVID (2016–2019) and Post-COVID (2020–2022) Era

Variable	Pre-COVID (2016–2019)			Post-COVID (2020–2022)		
	B	95% CI	p	B	95% CI	p
**Age, per year**	−0.047	−0.063 to −0.031	<0.001*	−0.058	−0.075 to −0.042	<0.001*
**Female sex (ref: male)**	+0.680	0.356–1.005	<0.001*	+1.193	0.858–1.528	<0.001*
**Race/Ethnicity (ref: White)**						
Black	+1.713	0.903–2.524	<0.001*	+3.901	3.117–4.685	<0.001*
Hispanic	+0.679	0.229–1.129	0.003*	+0.672	0.196–1.148	0.006*
Asian/Pacific Islander	−0.768	−1.881 to 0.344	0.176	+2.034	0.885–3.184	0.001*
Native American	−0.829	−2.096 to 0.438	0.200	+1.554	0.251–2.857	0.019*
Other	−0.027	−0.932 to 0.878	0.953	−0.325	−1.185 to 0.535	0.459
**Income quartile (ref: 0–25th percentile)**						
26th–50th percentile	−1.305	−1.727 to −0.883	<0.001*	+0.477	0.032–0.922	0.035*
51st–75th percentile	−0.533	−0.986 to −0.080	0.021*	+0.379	−0.097 to 0.856	0.118
76th–100th percentile	−0.348	−0.883 to 0.187	0.203	+0.766	0.207–1.325	0.007*
**Primary payer (ref: Medicare)**						
Medicaid	+0.673	0.203–1.143	0.005*	+1.509	1.011–2.006	<0.001*
Private insurance	+0.262	−0.165 to 0.690	0.229	+0.476	0.017–0.934	0.042*
Self-pay	−0.844	−1.645 to −0.042	0.039*	+0.307	−0.557 to 1.170	0.486
Other	+0.081	−0.834 to 0.996	0.862	+2.537	1.608–3.466	<0.001*
**Region (ref: Northeast)**						
Midwest	−2.660	−3.182 to −2.139	<0.001*	−2.419	−2.961 to −1.876	<0.001*

South	−1.468	−1.949 to −0.987	<0.001*	−2.293	−2.802 to −1.783	<0.001*
West	−2.740	−3.268 to −2.212	<0.001*	−2.400	−2.943 to −1.857	<0.001*
**Hospital teaching status (ref: rural)**						
Urban teaching	+2.282	0.817–3.747	0.002*	+1.632	0.156–3.109	0.030*
**Hospital bed size (ref: small)**						
Large	+0.802	0.175–1.429	0.012*	+1.613	0.986–2.240	<0.001*
**Urban–rural code (ref: central metro ≥1M)**						
Fringe metro ≥1M	+0.737	0.257–1.217	0.003*	−0.693	−1.183 to −0.202	0.006*
Metro 250k–999k	−1.156	−1.619 to −0.693	<0.001*	−0.553	−1.021 to −0.084	0.021*
Not metro/micropolitan	−0.470	−1.174 to 0.234	0.190	−1.775	−2.545 to −1.004	<0.001*
**Weekend admission**	+2.609	2.187–3.031	<0.001*	+1.770	1.352–2.187	<0.001*
**Elixhauser Comorbidity Index, per unit**	+0.461	0.440–0.481	<0.001*	+0.484	0.463–0.505	<0.001*

**p<0.05. B, unstandardized coefficient; CI, confidence interval. Notable: Black LOS disparity nearly doubled (+1.71 days pre-COVID vs +3.90 days post-COVID). Medicaid LOS increased from +0.67 to +1.51 days post-COVID. Asian/Pacific Islander and Native American patients non-significant pre-COVID but significantly longer LOS post-COVID.*
